# Three-dimensional sonography has satisfied accuracy for detecting rotator cuff tears

**DOI:** 10.3389/fsurg.2024.1411816

**Published:** 2024-05-15

**Authors:** Xin Wang, Wei Zhang, Jinlei Dong, Lianxin Li, Yuzhi Xiao, Fanxiao Liu

**Affiliations:** ^1^ Department of Anesthesia Operating Room, Shandong Provincial Hospital Affiliated to Shandong First Medical University, Jinan, Shandong, China; ^2^ Department of Orthopaedics, Shandong Provincial Hospital Affiliated to Shandong First Medical University, Jinan, Shandong, China

**Keywords:** shoulder pain, rotator cuff tears, sonography, diagnostic study, tear pattern, 3D

## Abstract

**Background:**

Rotator cuff injuries and tears are common causes of shoulder pain and dysfunction, necessitating accurate diagnostic methods to guide clinical decision-making. This study evaluates the diagnostic utility of three-dimensional (3D) shoulder sonography in identifying rotator cuff injury and tear patterns.

**Methods:**

A comprehensive search across seven electronic databases, which included Cochrane Library, Embase, PubMed, Cochrane Library, China Biology Medicine (CBM) database, CNKI, Wanfang, and VIP database. These databases were utilized to retrieve articles that assess the diagnostic value of 3D shoulder sonography for identifying rotator cuff injuries and tear patterns. The effectiveness of 3D shoulder sonography was assessed in terms of sensitivity, specificity, positive likelihood ratio (PLR), negative likelihood ratio (NLR), and diagnostic odds ratio (DOR). For each parameter, the 95% confidence intervals were calculated. Additionally, summary receiver operating characteristic curves (SROCs) were constructed, allowing for a comprehensive evaluation of diagnostic accuracy, which is reflected in the area under the SROC curve (AUC).

**Results:**

Screening of 8,508 identified nine literatures eligible for inclusion in the meta-analysis, encompassing a total of 366 patients. The analysis of detecting any rotator cuff tear revealed a sensitivity of 0.97 and specificity of 0.87, yielding a DOR of 90.03 and an AUC of 0.98. Furthermore, 3D shoulder sonography demonstrated satisfactory accuracy in detecting both full and partial-thickness rotator cuff tears (Sensitivity: 0.92 vs. 0.83, specificity: 0.94 vs. 097, and AUC: 0.96 vs. 0.95).

**Conclusion:**

This study indicates that three-dimensional sonography has satisfied accuracy for detecting rotator cuff tears.

## 1 Introduction

The rotator cuff is a vital anatomical component of the shoulder joint, serving a crucial function in enabling the intricate range of motion and stability essential for the upper limb. Comprised of four distinct tendons—the supraspinatus, infraspinatus, teres minor, and subscapularis—the rotator cuff serves as a dynamic stabilizer and motor unit, ensuring the functional integrity of the shoulder complex. Given its fundamental role, any pathology affecting the rotator cuff, particularly tears, can lead to debilitating pain, functional impairment, and a significant decrease in the patient's quality of life (1–3).

Rotator cuff tears are among the most common musculoskeletal disorders encountered in clinical practice (4), particularly among individuals engaged in sports and activities demanding repetitive overhead motions, such as baseball pitchers, swimmers, and weightlifters (5). While acute trauma can cause rotator cuff tears, the condition often develops progressively over time, primarily due to degenerative changes associated with aging, vascular insufficiency, and intrinsic tendon degeneration. Irrespective of the etiology, accurate and timely diagnosis of rotator cuff tears is of paramount importance in guiding appropriate treatment decisions and optimizing patient outcomes (6, 7). The size of rotator cuff tears is a critical factor in determining the appropriate course of clinical management, particularly regarding surgical intervention (7). Small tears may often respond well to conservative treatments, such as physical therapy and anti-inflammatory medications, while larger tears often necessitate surgical repair to restore function and alleviate pain. Accurate measurement of tear size is thus essential for determining the most suitable treatment approach (6, 8, 9).

Traditionally, the diagnosis of rotator cuff tears has relied on a combination of clinical evaluation, patient history, and imaging modalities, including magnetic resonance imaging (MRI) and magnetic resonance arthrography (MRA). These conventional techniques have undoubtedly contributed significantly to the understanding and management of rotator cuff pathology. However, they are not without their limitations. MRI, although considered the gold standard for soft tissue imaging, is associated with several drawbacks, including high cost, limited availability, long imaging times, and contraindications for patients with certain metallic implants or claustrophobia. MRA, on the other hand, requires joint injection of contrast material, which may be uncomfortable for patients and carries a risk of complications (2, 6, 8, 10). Furthermore, both MRI and MRA are characterized by the need for a static supine position during imaging, limiting their ability to assess the shoulder dynamically, particularly during provocative maneuvers that replicate the patient's symptoms (2, 8, 10, 11). This limitation can result in false-negative findings, as tears may only become apparent under specific loading conditions.

The development of high frequency matrix probes and dedicated software as three-dimensional imaging implemented the role of sonography. Recently, there has been an increasing interest in the utilization of sonography as an alternative diagnostic tool for the assessment of rotator cuff tears (12). Sonography offers several advantages, including real-time imaging, cost-effectiveness, lack of ionizing radiation (10, 13), and the ability to perform dynamic assessments, making it an appealing option for evaluating the shoulder joint. Moreover, the introduction of three-dimensional (3D) sonography technology has further expanded the potential of sonography in musculoskeletal imaging (14). 3D sonography provides volumetric data and multiplanar reconstructions (15), allowing for a more comprehensive evaluation of complex anatomical structures, such as the rotator cuff. This modality offers the ability to visualize the full extent of a tear, assess the relative position of structures, and even perform dynamic evaluations while the patient actively moves their shoulder (16). While 3D shoulder sonography holds promise for diagnosing of rotator cuff injuries and tear patterns, the existing body of evidence is dispersed across various studies and presents variable findings (16). This necessitates a comprehensive meta-analysis to synthesize the available data, determine the overall diagnostic accuracy of 3D sonography, and identify potential factors influencing its performance.

Therefore, the main goal of this study is to conduct a meta-analysis to evaluate the diagnostic efficacy of 3D sonography in identifying rotator cuff injuries and tear patterns by consolidating data from multiple studies. This includes evaluating the sensitivity, specificity, positive likelihood ratio (PLR), negative likelihood ratio (NLR), diagnostic odds ratio (DOR), summary receiver operating characteristic curves (SROCs), and the area under the SROC curve (AUC). Additionally, we will explore potential sources of heterogeneity, such as operator experience, patient demographics, and tear characteristics, to offer a comprehensive perspective on the present status of 3D sonography in diagnosing rotator cuff injury and tear patterns.

## 2 Materials and methods

This study adhered to the Preferred Reporting Items for a Systematic Review and Meta-analysis of Diagnostic Test Accuracy Studies (PRISMA-DTA) guidelines (17). Ethical approval and patient consent were not applicable since the analysis utilized data from previously published articles.

### 2.1 Inclusion and exclusion criteria

Clinical studies that assessed the diagnostic capabilities of 3D sonography in identifying individuals with rotator cuff injuries were considered for inclusion in this study. We specifically included literatures that furnished the requisite data for sensitivity and specificity calculations in the final analysis. In cases where duplicate data were found among multiple articles, the study with the most comprehensive data or the most recent publication date was selected.

Exclusion criteria consisted of the following: (1) Letters, reviews, editorials, and other non-original research; (2) Conference proceedings; (3) Animal trials; (4) Articles that did not provide the necessary data for sensitivity and specificity calculations.

### 2.2 Selection strategy

The systematic search for relevant articles was conducted using a comprehensive strategy. Seven scientific databases, which included Cochrane Library, Embase, PubMed, Cochrane Library, China Biology Medicine (CBM), CNKI, Wanfang, and VIP, were systematically queried. The search terms employed were “ultrasound” or “sonography”, in combination with “rotator cuff”, “supraspinatus”, “subscapular muscle”, “shoulder pain” or “subscapularis”. No language restrictions were applied throughout the identifying procedure. The final search update was performed on January 31, 2024. Two independent and blinded investigators, designated as investigator A and B, performed the initial screening and assessment of retrieved studies according to the specified criteria. Titles and abstracts of articles were reviewed, and articles that did not align with the study's focus were excluded. Subsequently, the remaining articles underwent a detailed review. Additionally, a manual screening of reference lists from pertinent articles, including review, meta-analysis, and included study, was conducted to identify any additional studies that may not have been identified through the database search.

### 2.3 Data extraction

To minimize potential bias, the same two independent investigators who conducted the initial screening also carried out data extraction. Each eligible article provided the following information: first author's name, publication year and journal, study origin, number of cases and shoulders, case demographics including age and gender, sonography equipment and probe, rotator cuff tear type and patterns, the gold standard used for diagnosis, and inclusion interval of study. In studies included in the statistical analysis, rotator cuff tear patterns (full- and partial-thickness) and sizes (small, medium, and large) were classified as true positive (TP), false positive (FP), true negative (TN), or false negative (FN) based on the gold standard's confirmed outcomes. These TP, FP, TN, and FN values were recorded in a standardized Excel spreadsheet by two additional blinded investigators working independently.

### 2.4 Quality assessment

To minimize potential bias, quality assessment of the included studies was performed using the QUADAS-2 tool (18, 19) by the same two independent investigators who conducted the initial screening. QUADAS-2 is the updated version of the QUADAS tool, which is recommended for meta-analyses to assess the risk of bias and the relevance of primary diagnostic accuracy studies. The QUADAS-2 tool comprises four main domains with a total of 11 points: patient selection, index test, reference standard, and flow and timing. Each domain is evaluated for risk of bias, and concerns regarding applicability are also addressed.

### 2.5 Statistical analysis

Statistical analyses were conducted using STATA software version 12.0 (StataCorp, College Station, TX). The presence of a threshold effect was evaluated in the use of using the Spearman correlation coefficient, while heterogeneity across studies was assessed through the I-square test. Heterogeneity was considered not significant if I-square was less than 50% (20–22). Publication bias was evaluated using Deeks’ Funnel Plot Asymmetry test (23). A two-sided *p* < .05 was considered as significant for the analyses of threshold effect, heterogeneity, and publication bias. Meta regression was performed according to journal of publication, origin of study, number of cases, sonography probe, the gold standard used for diagnosis, inclusion interval of study, and QUADAS-2 scores. To quantitatively assess the diagnostic performance of 3D shoulder sonography in identifying rotator cuff tears and patterns, a random-effects model was employed to compute combined outcome estimates, which encompassed sensitivity, specificity, PLR, negative NLR, and DOR, accompanied by a corresponding 95% confidence interval (CI). Additionally, a SROC was constructed, where sensitivity was plotted on the x-coordinate and 1-specificity on the y-coordinate (24). The AUC was determined to assess the overall diagnostic accuracy of 3D shoulder sonography.

## 3 Results

### 3.1 Selection flow

**Figure 1:**
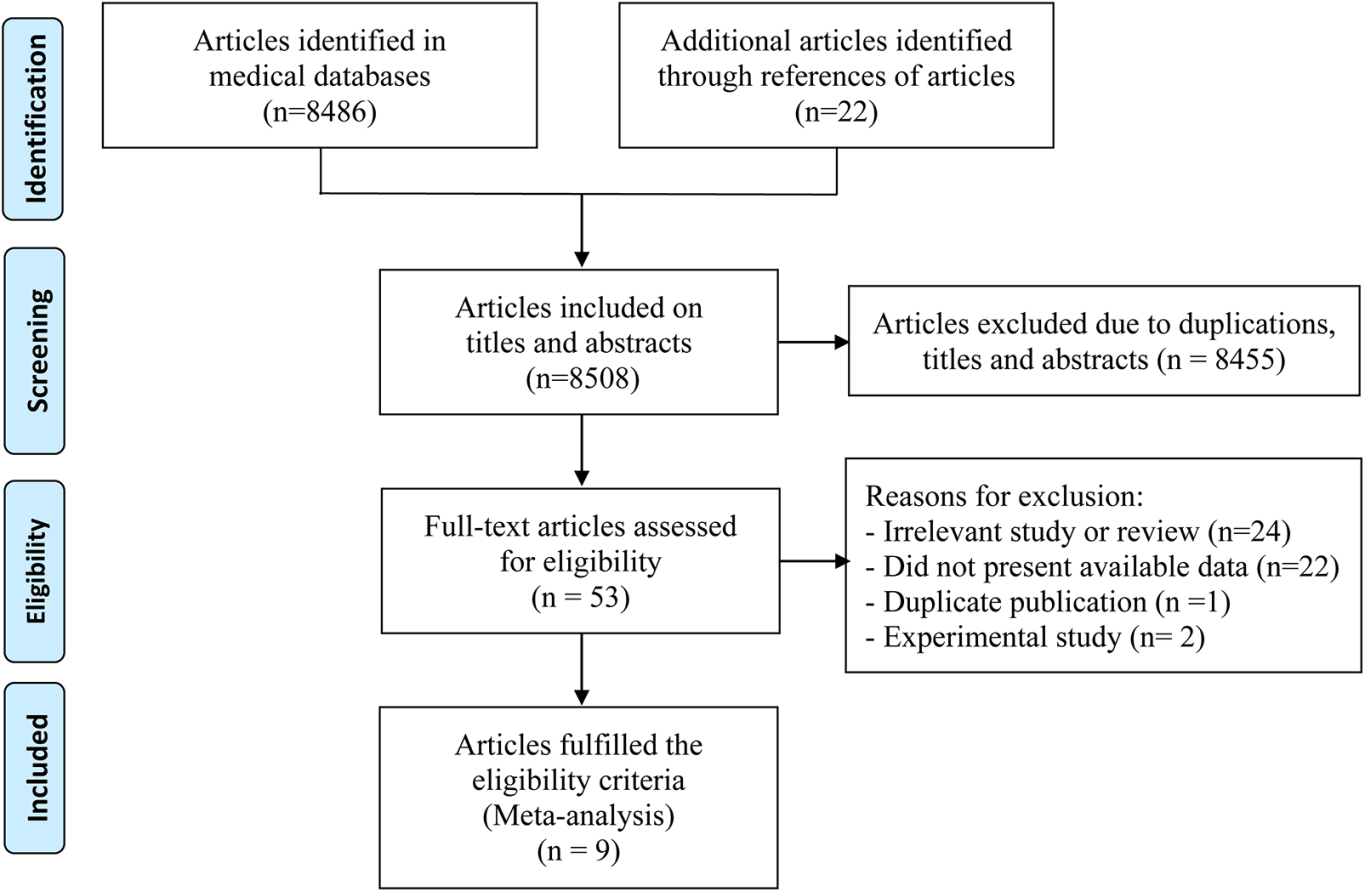
Flow diagram of the literature search and selection process.

Initially, 8,486 records were identified through the electronic database search, with an additional 22 records discovered in the reference lists of relevant studies found within the databases. Among the 8,486 records, 8,455 were excluded due to duplication or irrelevance based on titles and abstracts. From the 53 articles resulting from this selection process, one was a duplicate publication, two were referred to as experimental studies, and 22 had insufficient data for sensitivity and specificity calculations, leading to their exclusion. Following a rigorous selection process, a total of 9 articles (16, 25–32), involving 366 cases, were incorporated into the meta-analysis. A comprehensive overview of the article search and study selection process was show in Figure 1.

### 3.2 Basic information of the included studies

These included literatures were published over the span of 2000–2023, with sample sizes ranging from 25 to 52. A total of nine studies examined the utility of 3D shoulder sonography for detecting various types of rotator cuff tears, including any, full-thickness, and partial-thickness tears. Additionally, three studies specifically focused on identifying full-thickness rotator cuff tears according to tear size. Across all the studies included in our analysis, the gold standard for diagnosing rotator cuff tears was consistently arthroscopy or surgery. It is worth noting that some studies shared overlapping participants and provided data for different subgroup analyses. Basic information of the included studies is provided in Table 1. To ensure accuracy, two investigators independently extracted and verified all diagnostic original data from the original literatures, as outlined in Table 2.

**Table 1:** Basic information of the included studies.

Study, year	Country	Formal publish of journal	No. of subjects	No. of shoulders	Sex (M/F)	Age (range, yrs)	Sonography equipment	Sonography probe	Gold standard	Inclusion interval
Wallny et al. (32)	Germany	Ultraschall in Medicine	25	25	15/10	Mean 55	Ultraschallgerät sonoline SL	7.5–8.5 MHz	Arthroscopy, MRI	NA
Wallny et al. (31)	Germany	Ultrasound in Medicine and Biology	40	40	25/15	38–79, mean 54	Kretztechnik 530D	10 MHz	Arthroscopy, MRI	NA
Kang et al. (30)	Korea	Skeletal Radiology	50	50	32/18	22–78 (mean 55.6)	General electronic	8–15 MHz	Arthroscopy, MRI	2007.02–2008.08
Co et al. (29)	Canada	Canadian Association of Radiologists Journal	49	49	NA	NA	GE healthcare	9–12 MHz	Arthroscopy, MRI	NA
Hayter et al. (28)	America	Journal Ultrasound Medicine	42	42	25/17	18–83 (mean 60.2)	Philips broadband VL	13–15 MHz	2D-3D	NA
Gong et al. (27)	China	Chinese Journal of Geriatrics	30	30	18/12	50–70	GE logic E9	8–15 MHz	Arthroscopy	2012.03–2012.07
Jiang et al. (26)	China	Journal of Practical Hand Surgery	46	46	28/18	24–71, mean34	GE logic E9	8–15 MHz	Arthroscopy, MRI	2012.08–2014.01
Zhang et al. (25)	China	Chinese Journal Ultrasound Medicine	32	32	19/13	Mean 57	Philips iU elite	5–13 MHz	Arthroscopy, MRI	2014.9–2016.3
Chen et al. (16)	China	European Radiology	52	52	28/24	Mean 62.65	Philips iU elite; Samsung (RS85), LV3–14A	3–14 MHz	Arthroscopy	2021.2–2022.5

M, male; F, female; NA, not available; D, dimensional.

**Table 2:** The results of quality assessment of the included studies.

Study, year	1	2	3	4	5	6	7	8	9	10	11	QUADAS-2
Wallny et al. (32)	U	Y	Y	U	Y	Y	Y	Y	Y	Y	Y	9
Wallny et al. (31)	U	Y	Y	U	Y	Y	Y	Y	Y	Y	Y	9
Kang et al. (30)	Y	Y	Y	Y	Y	Y	Y	Y	Y	Y	Y	11
Co et al. (29)	U	Y	Y	Y	Y	Y	Y	Y	Y	Y	Y	10
Hayter et al. (28)	U	Y	Y	Y	Y	Y	Y	Y	Y	Y	Y	10
Gong et al. (27)	Y	Y	Y	U	Y	Y	Y	Y	Y	Y	Y	10
Jiang et al. (26)	Y	Y	Y	U	Y	Y	Y	Y	Y	Y	Y	10
Zhang et al. (25)	Y	Y	Y	U	Y	Y	Y	Y	Y	Y	Y	10
Chen et al. (16)	Y	Y	Y	Y	Y	Y	Y	Y	Y	Y	Y	11

1. Was a consecutive or random sample of patients enrolled?

2. Was a case-control design avoided?

3. Did the study avoid inappropriate exclusions?

4. Were the index test results interpreted without knowledge of the results of the reference standard?

5. If a threshold was used, was it pre-specified?

6. Is the reference standard likely to correctly classify the target condition?

7. Were the reference standard results interpreted without knowledge of the results of the index test?

8. Was there an appropriate interval between index test (s) and reference standard?

9. Did all patients receive a reference standard?

10. Did all patients receive the same reference standard?

11. Were all patients included in the analysis?

Y yes, N no, U unclear, *presents different study.

### 3.3 Quality assessment

According to the standard of QUADAS-2 and following a rigorous assessment process, two studies attained 9 points, five achieved 10 points, and the remaining 2 got 11 points, as shown in Table 3.

**Table 3:** Diagnosis accuracy data of 3D shoulder US for rotator cuff tears.

Study, year	Total	True positive	False positive	False negative	True negative	Lesion type
Wallny et al. (32)	25	12	2	0	11	RCT
Wallny et al. (31)	40	14	2	3	21	RCT
Kang et al. (30)	50	43	0	2	5	RCT
Hayter et al. (28)	42	24	9	3	6	RCT
Co et al. (29)	54	21	0	1	32	RCT
Gong et al. (27)	30	30	0	0	0	RCT
Jiang et al. (26)	46	46	0	0	0	RCT
Wallny et al. (32)	25	4	0	0	21	FT-RCT
Wallny et al. (31)	40	18	1	0	21	FT-RCT
Kang et al. (30)	50	35	1	5	9	FT-RCT
Co et al. (29)	54	12	0	0	42	FT-RCT
Hayter et al. (28)	42	11	0	2	29	FT-RCT
Gong et al. (27)	30	21	1	2	6	FT-RCT
Jiang et al. (26)	46	36	1	3	6	FT-RCT
Zhang et al. (25)	32	19	1	2	10	FT-RCT
Chen et al. (16)	52	52	0	0	0	FT-RCT
Wallny et al. (32)	25	2	1	1	21	PT-RCT
Wallny et al. (31)	40	3	3	2	32	PT-RCT
Kang et al. (30)	50	2	5	3	40	PT-RCT
Co et al. (29)	54	9	1	1	28	PT-RCT
Hayter et al. (28)	42	12	10	2	20	PT-RCT
Gong et al. (27)	30	6	2	1	21	PT-RCT
Jiang et al. (26)	46	6	3	1	36	PT-RCT
Zhang et al. (25)	32	10	2	1	19	PT-RCT
Kang et al. (30)	50	23	0	12	15	Size patterns
Chen et al. (16)	52	43	9	0	0	Size patterns
Kang et al. (30)	35	2	0	0	33	S-FT-RCT
Zhang et al. (25)	17	1	2	0	14	S-FT-RCT
Kang et al. (30)	35	14	0	4	16	M-FT-RCT
Zhang et al. (25)	17	5	1	2	9	M-FT-RCT
Kang et al. (30)	35	7	0	8	19	L-FT-RCT
Zhang et al. (25)	17	9	1	0	7	L-FT-RCT
Chen et al. (16)	52	4	1	0	47	L-FT-RCT

RCT, rotator cuff tear; FT, full-thickness; PT, partial-thickness; S, small; M, media; L, large.

### 3.4 Diagnostic value for any tear

**Figure 2:**
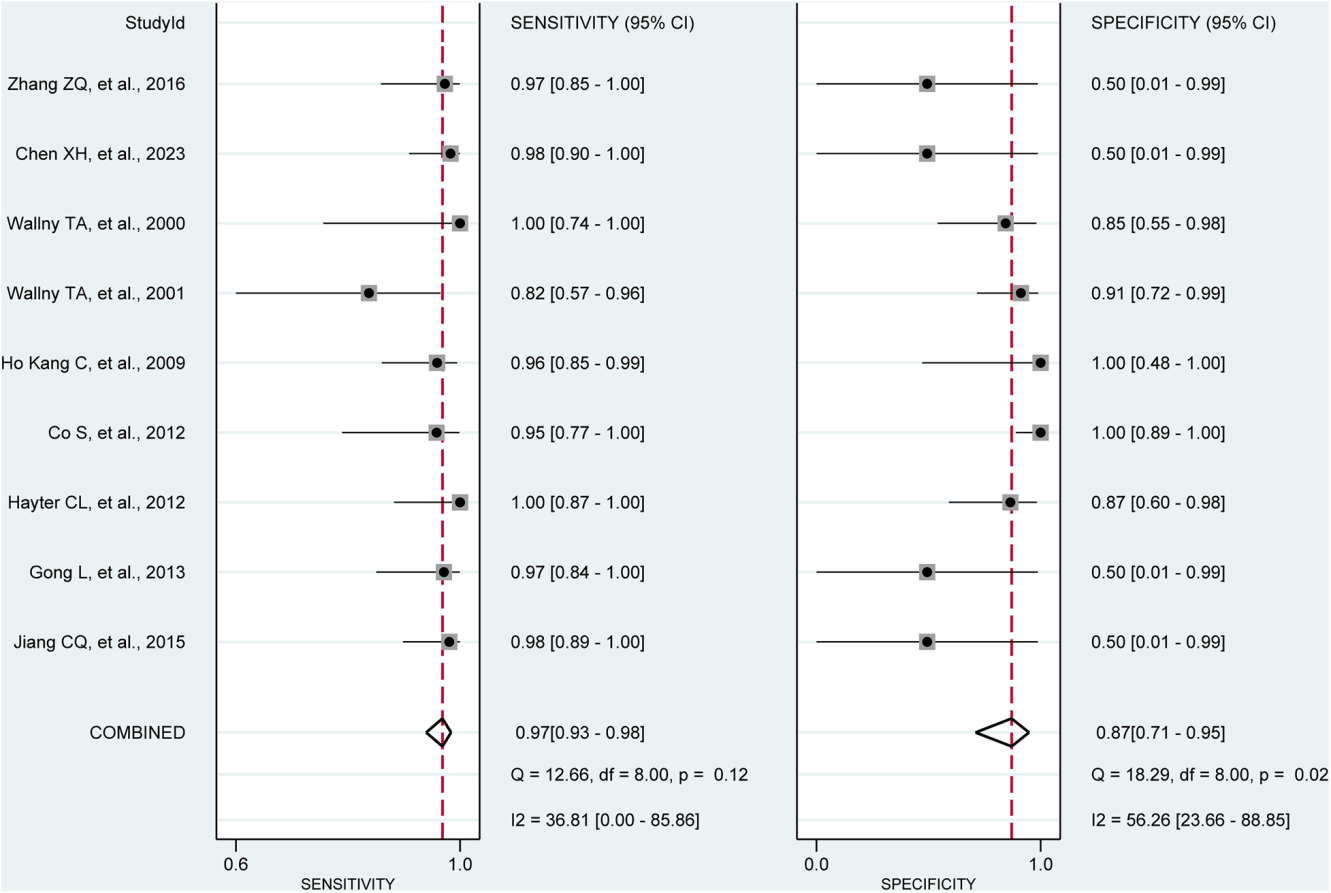
Forest plots of the pooled sensitivity and specificity to diagnose any rotator cuff tear with the corresponding 95% confidence region. Three-dimensional sonography for the diagnosis of patients with any rotator cuff tear (nine studies, the pooled sensitivity = 0.97, *I*^2 ^= 36.81%; the pooled specificity = 0.87, *I*^2 ^= 56.26%). CI, confidence interval.

**Figure 3:**
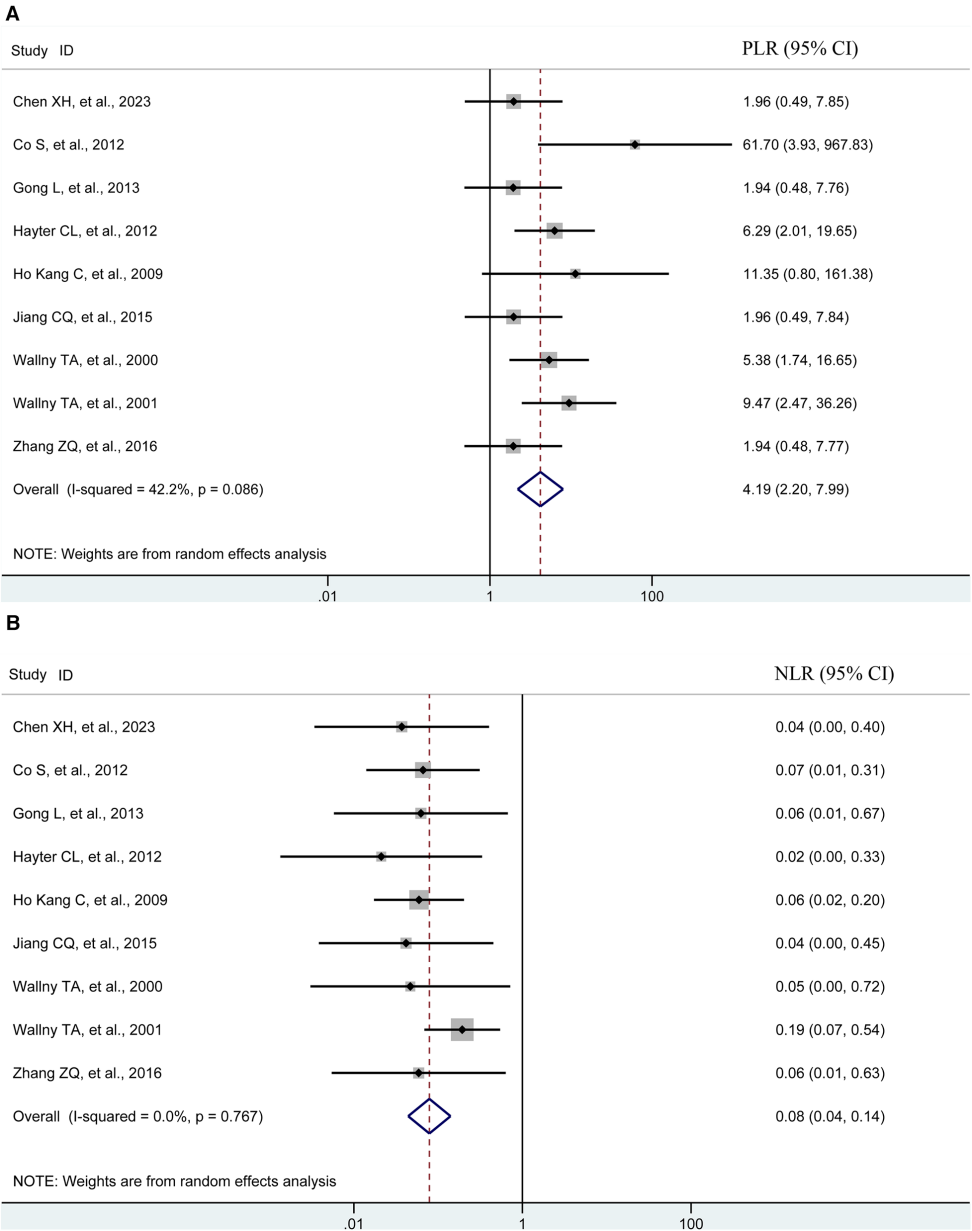
Forest plots of the pooled PLR (**A**) and NLR (**B**) to diagnose any rotator cuff tear with the corresponding 95% confidence region. The diamond in the right of the central vertical line represents a higher PLR or NLR to diagnose any rotator cuff tear. NLR, negative likelihood ratio; PLR, positive likelihood ratio; CI, confidence interval.

**Figure 4:**
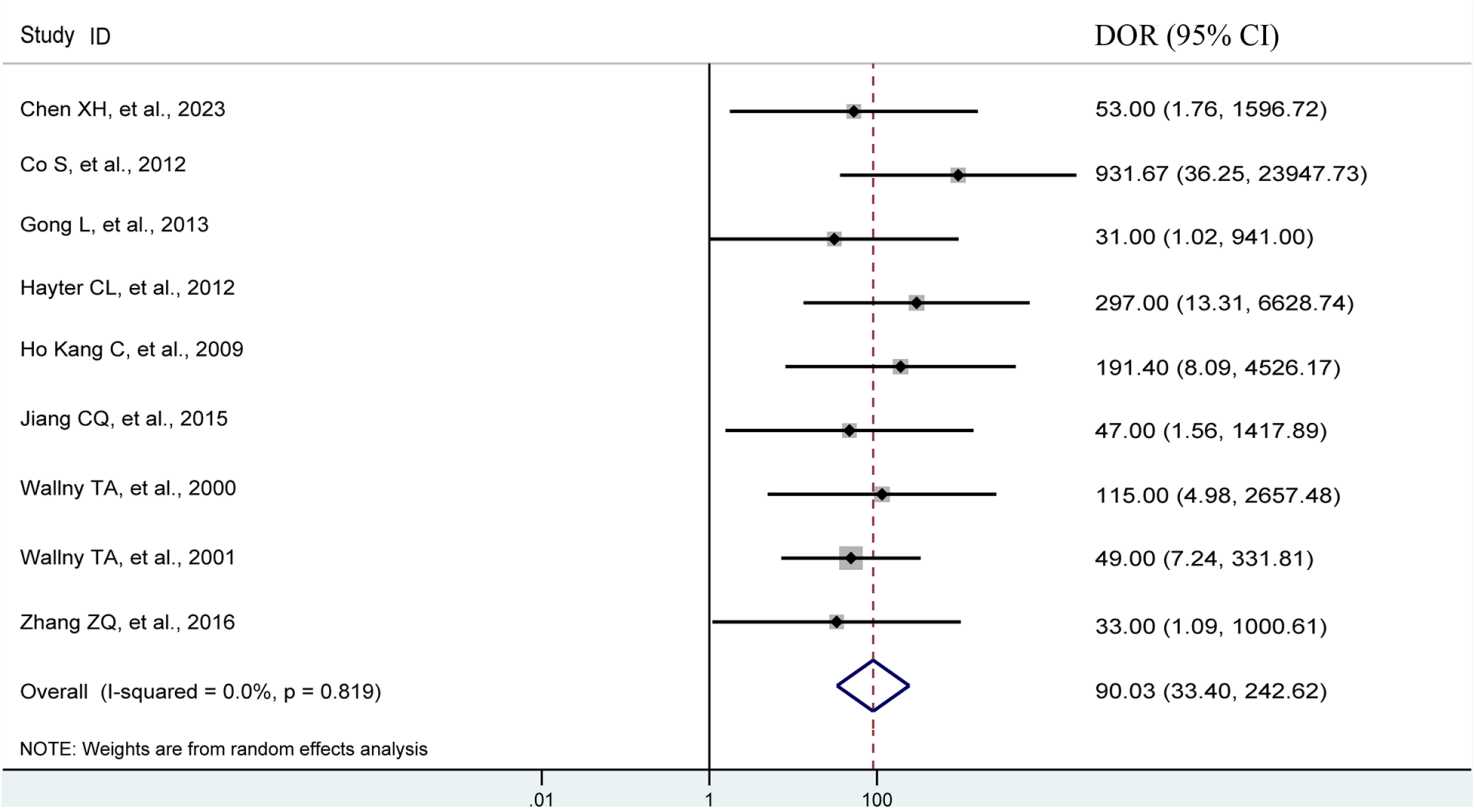
Forest plots of the pooled diagnostic odds ratio (DOR) to diagnose any rotator cuff tear with the corresponding 95% confidence region. The diamond in the right of the central vertical line represents a higher DOR to diagnose any rotator cuff tear. DOR, diagnostic odds ratio; CI, confidence interval.

**Figure 5:**
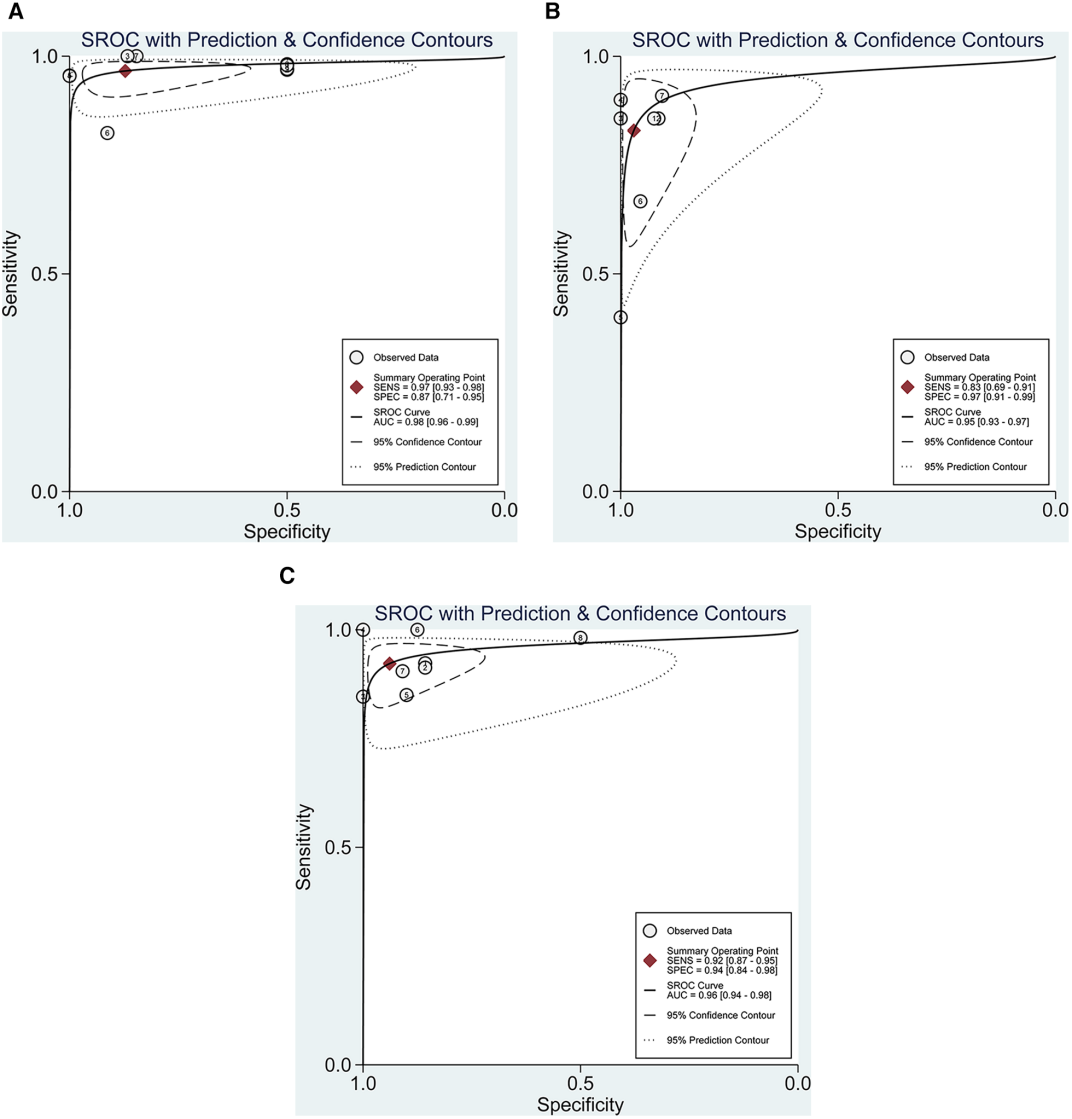
Summarized receiver operating characteristic curve (sROC) for the diagnosis of any (**A**), partial- (**B**), and full-thickness (**C**) rotator cuff tears with the corresponding 95% confidence region.

**Figure 6:**
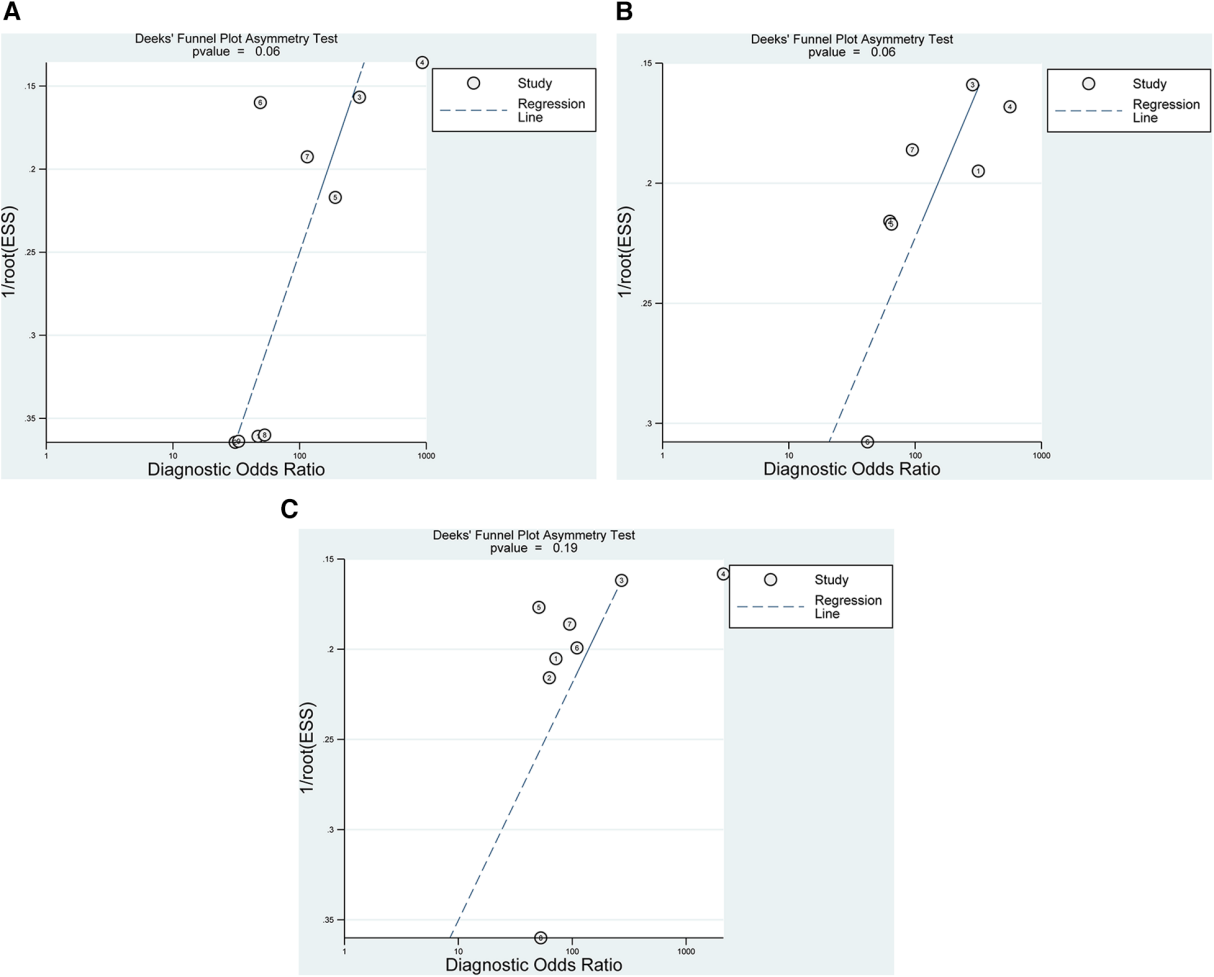
Graphical display of the results of deek's test for publication bias for the diagnosis of any (**A**), partial- (**B**), and full-thickness (**C**) rotator cuff tears (the *p*-value is 0.06, 0.06, and 0.19, respectively).

Pooling the results from 9 studies, which collectively involved 371 shoulders, we evaluated the diagnostic value of 3D sonography in identifying any rotator cuff tear. Our analysis revealed a sensitivity of 0.97 (95% CI, 0.93–0.98), a specificity of 0.87 (95% CI, 0.71–0.95), a PLR of 4.19 (95% CI, 2.20–7.99), an NLR of 0.08 (95% CI, 0.04–0.14), and a DOR of 90.03 (95% CI, 33.40–242.62) as depicted in Figures 2–4. Importantly, we did not observe a threshold effect in the analysis (*p* = 0.182). The AUC of the SROC curve was 0.98 (95% CI, 0.96–0.99) as illustrated in Figure 5A. Furthermore, the results of Deeks’ Funnel Plot Asymmetry test indicated no publication bias when assessing the performance of 3D sonography in identifying any rotator cuff tear, as seen in Figure 6A.

### 3.5 Detection of partial-thickness tears

**Figure 7:**
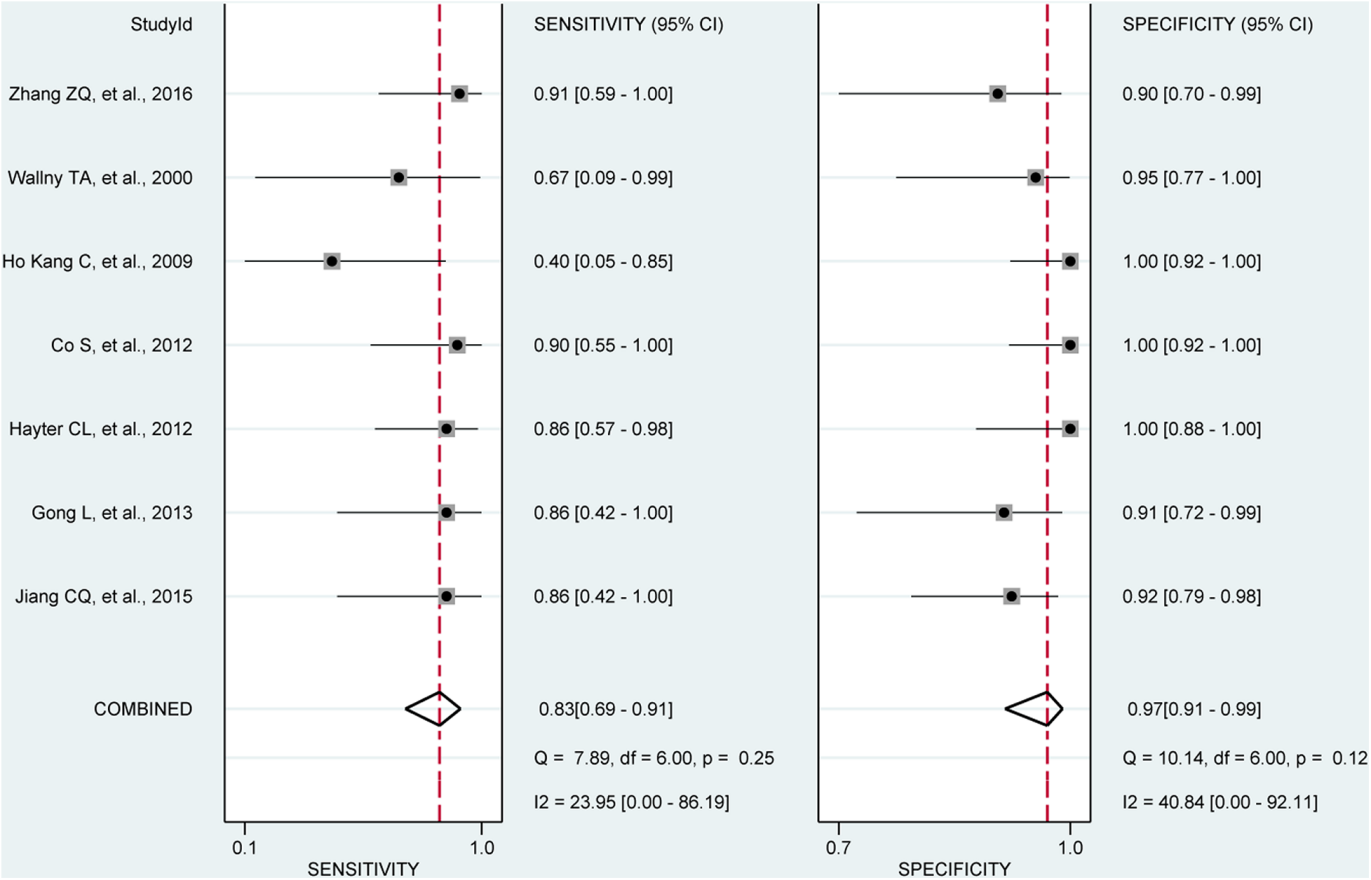
Forest plots of the pooled sensitivity and specificity to diagnose partial-thickness rotator cuff tears with the corresponding 95% confidence region. Three-dimensional sonography for the diagnosis of patients with partial-thickness rotator cuff tear (seven studies, the pooled sensitivity = 0.83, *I*^2 ^= 23.95%; the pooled specificity = 0.97, *I*^2 ^= 40.84%). CI, confidence interval.

In our analysis of seven studies including 279 shoulders, we assessed the diagnostic value of 3D sonography in detecting partial-thickness rotator cuff tears. No threshold effect was observed (*p*-value = 0.0513). Our findings indicated a sensitivity of 0.83 (95% CI, 0.69–0. 91) and specificity of 0.97 (95% CI, 0.91–0.99), as depicted in Figure 7. We also calculated a PLR of 13.62 (95% CI, 7.24–25.62), NLR of 0.23 (95% CI, 0.12–0.46), and DOR of 100.60 (95% CI, 34.53–293.05), as shown in Supplementary Figures S1A,B,S2. The AUC of the SROC curve was 0.95 (95% CI, 0.93–0.97), illustrated in Figure 5B. Furthermore, the results of Deeks’ Funnel Plot Asymmetry test suggested the absence of publication bias (*p* = 0.06, Figure 6B).

### 3.6 Identification of full-thickness tears

**Figure 8:**
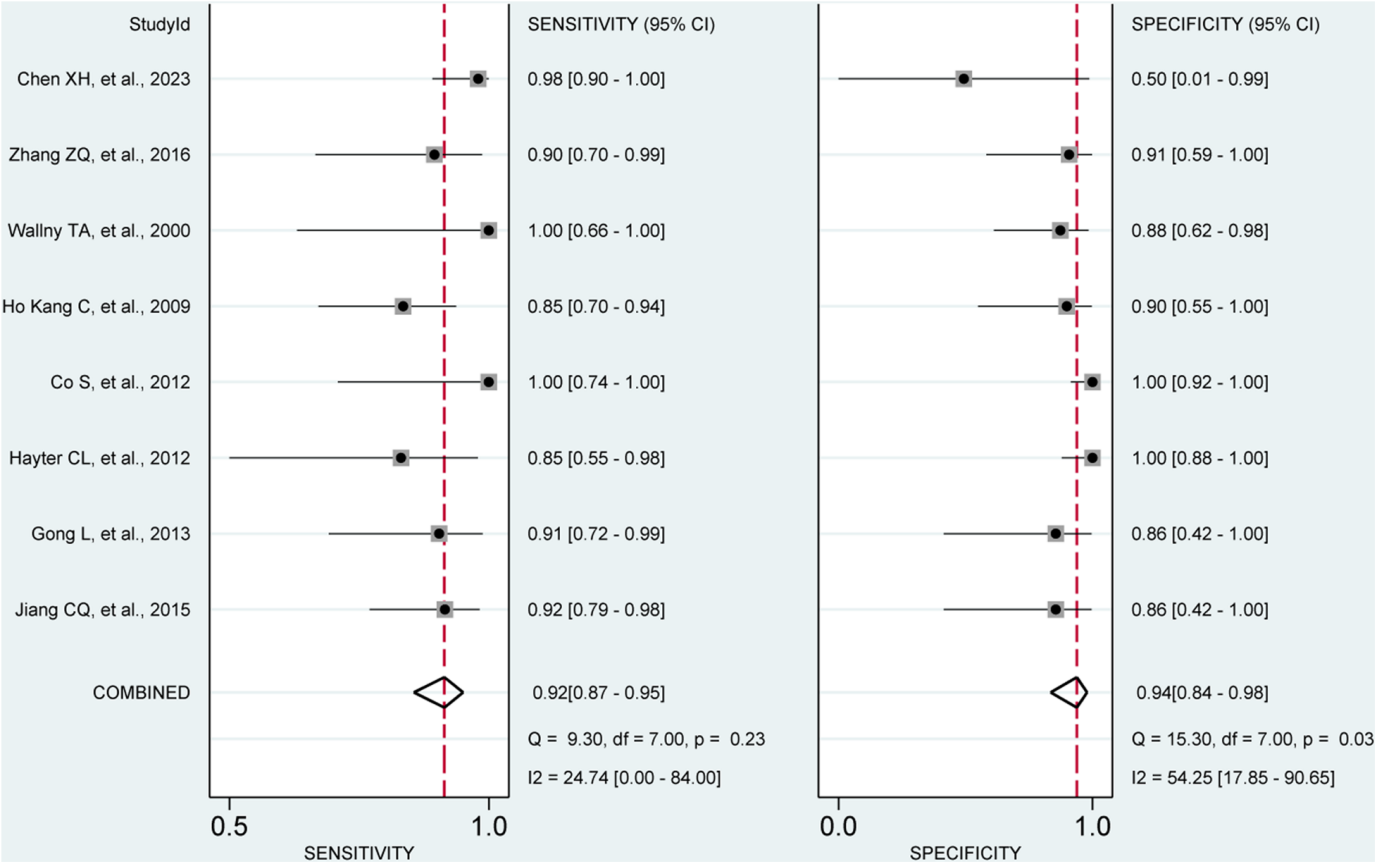
Forest plots of the pooled sensitivity and specificity to diagnose full-thickness rotator cuff tears with the corresponding 95% confidence region. Three-dimensional sonography for the diagnosis of patients with full-thickness rotator cuff tear (eight studies, the pooled sensitivity = 0.92, *I*^2 ^= 24.74%; the pooled specificity = 0.94, *I*^2 ^= 54.25%). CI, confidence interval.

Our analysis of eight studies involving 331 shoulders evaluated the diagnostic value of 3D sonography for identifying full-thickness tears, which indicated a sensitivity of 0.92 (95% CI 0.87–0.95) and a specificity of 0.94 (95% CI, 0.84–0.98), as depicted in Figure 8. We also calculated a PLR of 7.50 (95% CI, 36.80–263.32), NLR of 0.12 (95% CI, 0.07–0.19), and DOR of 98.44 (95% CI, 36.80–263.32), as shown in Supplementary Figures S3A,B,S4. Similar to the other analyses, no threshold effect was observed (*p* = 0.908). The AUC of the SROC curve was 0.96 (95% CI, 0.94–0.98), as illustrated in Figure 5C. Additionally, the results of Deeks’ Funnel Plot Asymmetry test indicated no publication bias (*p*-value = 0.19, Figure 6C).

### 3.7 Detection of full-thickness tear size and patterns

An assessment of the diagnostic value of the 3D sonography for identifying full-thickness tear size and patterns was conducted based on data from only 3 studies, involving 104 patients. This analysis demonstrated a sensitivity of 0.60, 0.95, and 0.91 for small, medium, and large full-thickness tears, respectively, along with a specificity of 1.00, 0.81, and 0.90 for small, medium, and large full-thickness tears, respectively. However, due to the limited number of studies involving a small patient population, these results should be interpreted cautiously.

## 4 Discussion

Rotator cuff injury is a prevalent cause of shoulder pain and impairment, affecting individuals across different age groups and activity levels. The accurate diagnosis of rotator cuff tears is crucial for guiding appropriate treatment decisions and optimizing patient outcomes. Recently, 3D shoulder sonography has emerged as a diagnostic tool with the potential to improve the accuracy and efficiency of diagnosing these tears. Our meta-analysis, incorporating data from multiple studies, demonstrates that 3D shoulder sonography exhibits a commendable sensitivity of 0.97 with the range from 0.93 to 0.98 and specificity of 0.87 with the range from 0.71 to 0.95 in the detection of any rotator cuff tear. One previous meta-analysis (10) performed by our team which included a total of 144 studies involving 14,059 patients (14,212 shoulders) revealed that MRA had the highest sensitivity and specificity (0.89 and 0.91, respectively). Meanwhile, MRI had better performance than US (sensitivity: 0.84 vs. 0.81; specificity: 0.86 vs. 0.82) for the detection of any tear. From the above data, it can be seen that, three-dimensional sonography has satisfied accuracy for detecting rotator cuff tears.

The introduction of 3D tomographic techniques in sonography imaging has made significant contributions to reducing operator subjectivity. The prospective study by Chen et al. (16) emphasizes the clinical use of 3D sonography in the morphologic assessment of rotator cuff injuries and tears patterns. The ability to assess tear size, muscle involvement, and fatty infiltration using 3D sonography can have significant implications for clinical decision-making. Differentiating between partial- and full-thickness tears, as well as evaluating muscle quality, are crucial factors in determining the suitable treatment strategy. Additionally, the study by Xu et al. (33) investigating longitudinal changes in supraspinatus muscle volume and intramuscular fatty infiltration following arthroscopic rotator cuff repair underscores the potential utility of 3D sonography in monitoring postoperative outcomes. This longitudinal assessment can aid in evaluating the success of surgical interventions and guiding rehabilitation strategies. The utilization of 3D sonography not only provides essential diagnostic information but also offers a comprehensive understanding of the relative position of anatomical sections (29). This multi-dimensional perspective minimizes operator-dependent variability and facilitates precise evaluations of complex anatomical situations associated with rotator cuff tears. Nevertheless, it's worth noting that the diagnostic precision of 3D sonography can be influenced by the operator's experience and the precision measure of sonography machines, particularly those equipped with high-resolution capabilities (28). Furthermore, the ability to visualize the tear in three dimensions provides a more comprehensive understanding of its size, shape, and characteristics, contributing to accurate diagnosis and treatment planning.

Numerous studies have reported sensitivity and specificity values for 3D sonography ranging from 0.77 to 1.00 and 0.50 to 0.90, respectively (9, 16, 28–30, 32). Kijima et al. (34) conducted pioneering research quantitatively demonstrating the reproducibility of 3D sonography in assessing the configuration of tear lesions, achieving a concordance rate of 0.91. Additionally, experimental studies indicated that 3D sonography is particularly effective in diagnosing rotator cuff injuries, with sensitivities of 0.77 and 0.85 for 3D and 2D sonography, respectively. One previous study by Teng et al. (9) provides valuable insights into the effectiveness of 3D sonography in identifying rotator cuff injuries. This study, along with other research, highlights the diagnostic precision and dependability of 3D sonography as a non-invasive and radiation-free imaging method. The results suggest that 3D sonography has a high sensitivity and specificity in detecting rotator cuff tears, making it a valuable tool in clinical practice. One of the significant advantages of 3D sonography is its real-time imaging capability. This feature allows clinicians to assess the shoulder joint dynamically, particularly during different movements, which can aid in the identification of tears that may not be apparent in static images (33). Our present study, involving 366 3D sonography examinations for the type of rotator cuff tear that confirmed by comprehensive diagnosis based on MRI, surgical operations, or shoulder arthroscopy, revealed a sensitivity and specificity values of 0.92 and 0.94 for full-thickness rotator cuff tears and 0.83 and 0.97 for partial-thickness tears, respectively. One previous meta-analysis performed by our team (10) which included a total of 144 studies involving 14,059 patients (14,212 shoulders) revealed that MRA had the highest sensitivity and specificity (0.95 and 0.96, respectively). MRI had a higher sensitivity than US (0.91 vs. 0.87) and a similar specificity (0.88 vs. 0.88) for the detection of full-thickness tear. Where available, the results for partial-thickness tears were similar to the detection of full-thickness tears. From the above data, it can be seen that, three-dimensional sonography has satisfied accuracy for detecting partial- or full-thickness rotator cuff tears.

The meta-analysis by Lee et al. (35) comparing 3D shoulder sonography with 2D conventional MR arthrography highlights the potential benefits of 3D sonography in diagnosing rotator cuff tears. While MR arthrography is considered one of the gold standards for diagnosing these tears, it is an invasive process that requires the injection of contrast agents. The study suggests that 3D sonography may offer comparable diagnostic accuracy without the need for invasive measures. Furthermore, the meta-analysis by Li et al. (6) focusing on articular-sided partial-thickness rotator cuff tears demonstrates that magnetic resonance arthrography (MRA) improves sensitivity compared to traditional MRI. However, MRA also involves the use of contrast agents and can be costlier and less accessible. In contrast, 3D shoulder sonography provides an alternative non-invasive option with potential benefits in terms of cost-effectiveness and accessibility.

Given the relevance of rotator cuff tear size in determining the appropriate course of surgical intervention, accurate classification of rotator cuff tears as small, medium, large, or massive is crucial. External rotation tests, especially in the shortened position, have shown promise in predicting tear size, particularly for the infraspinatus muscle (36). While limited studies have directly compared the accuracy of sonography and MRI in quantifying tear size, it is imperative to acknowledge that our findings revealed a sensitivity of 0.60, 0.95, and 0.91 for small, medium, and large full-thickness rotator cuff tears, respectively, along with a specificity of 1.00, 0.81, and 0.90 for small, medium, and large full-thickness rotator cuff tears, respectively. One study (16) revealed that the evaluation accuracy of three-dimensional sonography for tear pattern was higher than that of MRI (75.0%, 95% CI: 62.8%–87.2%). From the above data, it can be seen that both three-dimensional sonography and MRI has relative low accuracy for detecting tears patterns. These findings are derived from the limited data of only three included studies, and additional research is necessary to establish conclusive findings in this regard.

Despite the promising findings, it is crucial to recognize the limitations of 3D shoulder sonography. Operator dependence is one significant limitation, as obtaining high-quality images requires expertise in sonography techniques. Compared to 2D ultrasound, 3D ultrasound examination reduces operator dependent variability, but compared to MRI, 3D ultrasound still has operator dependent variation. Additionally, the effectiveness of 3D sonography can be influenced by patient factors such as body habitus and shoulder anatomy. Future research should focus on standardizing protocols for 3D shoulder sonography and training clinicians to ensure consistent and reliable results. Addressing these limitations will be crucial in maximizing the clinical utility of 3D sonography for diagnosing rotator cuff tears.

While our meta-analysis has yielded promising results, it is essential to acknowledge several limitations. First, methodological variability, including the use of various linear arrays for sonography, could introduce variations in the findings drawn from calculations. Various linear arrays indicate different frequencies of sonography, and our previous study (10) confirmed that high-frequency sonography have better performance than that of low-frequency sonography, which may have an impact on the results. Additionally, the diagnostic standards and categorization of tear patterns of rotator cuff were not standardized across all included studies, contributing to heterogeneity in our findings. Furthermore, demographic information about the participants, including gender and age, was not consistently available in the included studies. The limited availability of data for certain evaluation, such as small rotator cuff tears, could potentially lead to an overestimation or underestimation of relevant data.

## 5 Conclusion

The meta-analysis of existing studies highlights the potential of 3D shoulder sonography as a valuable tool for diagnosing any or full-thickness rotator cuff tears. However, it is crucial to recognize its limitations in detecting partial-thickness tears. Further research and large-scale trials are needed to validate and refine the diagnostic protocols involving 3D sonography, aiming to enhance its diagnostic capabilities across all forms of rotator cuff tears. The integration of 3D sonography into clinical practice has the potential to revolutionize the assessment of rotator cuff injuries, ultimately improving patient care and outcomes in the field of sports medicine and musculoskeletal health.

## Data Availability

The original contributions presented in the study are included in the article/**Supplementary Material**, further inquiries can be directed to the corresponding authors.

## Supplementary material

Supplementary Figure S1 Forest plots of the pooled PLR (**A**) and NLR (**B**) to diagnose partial-thickness rotator cuff tears with the corresponding 95% confidence region. The diamond in the right of the central vertical line represents a higher PLR or NLR to diagnose any rotator cuff tear. NLR, negative likelihood ratio; PLR, positive likelihood ratio; CI, confidence interval.

Supplementary Figure S2 Forest plots of the pooled diagnostic odds ratio (DOR) to diagnose partial-thickness rotator cuff tears with the corresponding 95% confidence region. The diamond in the right of the central vertical line represents a higher DOR to diagnose any rotator cuff tear. DOR, diagnostic odds ratio; CI, confidence interval.

Supplementary Figure S3 Forest plots of the pooled PLR (**A**) and NLR (**B**) to diagnose full-thickness rotator cuff tear with the corresponding 95% confidence region. The diamond in the right of the central vertical line represents a higher PLR or NLR to diagnose any rotator cuff tear. NLR, negative likelihood ratio; PLR, positive likelihood ratio; CI, confidence interval.

Supplementary Figure S4 Forest plots of the pooled diagnostic odds ratio (DOR) to diagnose full-thickness rotator cuff tears with the corresponding 95% confidence region. The diamond in the right of the central vertical line represents a higher DOR to diagnose any rotator cuff tear. DOR, diagnostic odds ratio; CI, confidence interval.
