# Supplementary figures and images for: Three-dimensional sonography has satisfied accuracy for detecting rotator cuff tears
Source: Front Surg. 2024 May 15;11:1411816. doi: 10.3389/fsurg.2024.1411816 (PMC11133732; doi:10.3389/fsurg.2024.1411816)

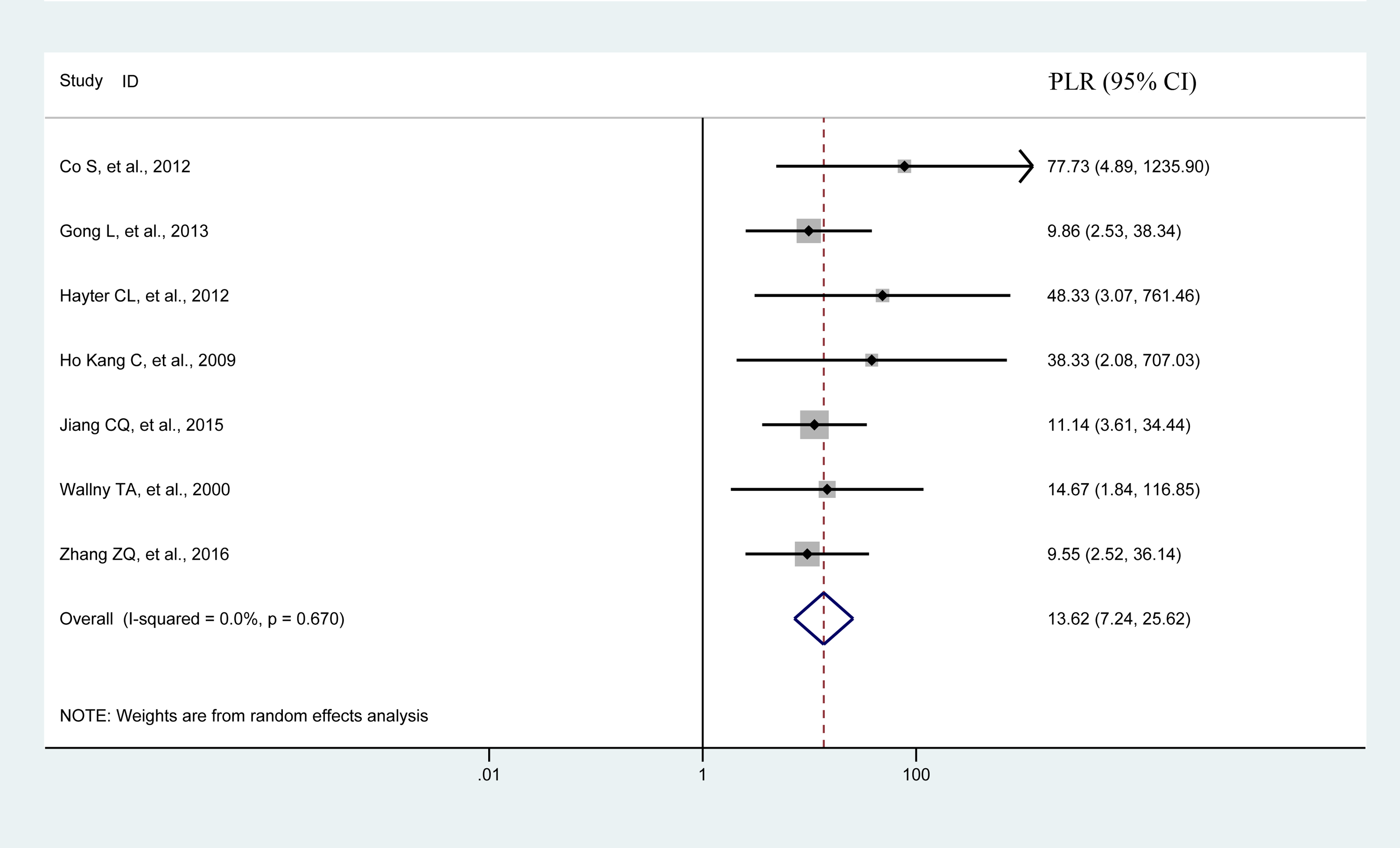

Supplement: Supplementary Figure S1 — Forest plots of the pooled PLR (A) and NLR (B) to diagnose partial-thickness rotator cuff tears with the corresponding 95% confidence region. The diamond in the right of the central vertical line represents a higher PLR or NLR to diagnose any rotator cuff tear. NLR, negative likelihood ratio; PLR, positive likelihood ratio; CI, confidence interval. [file Image1.tiff]

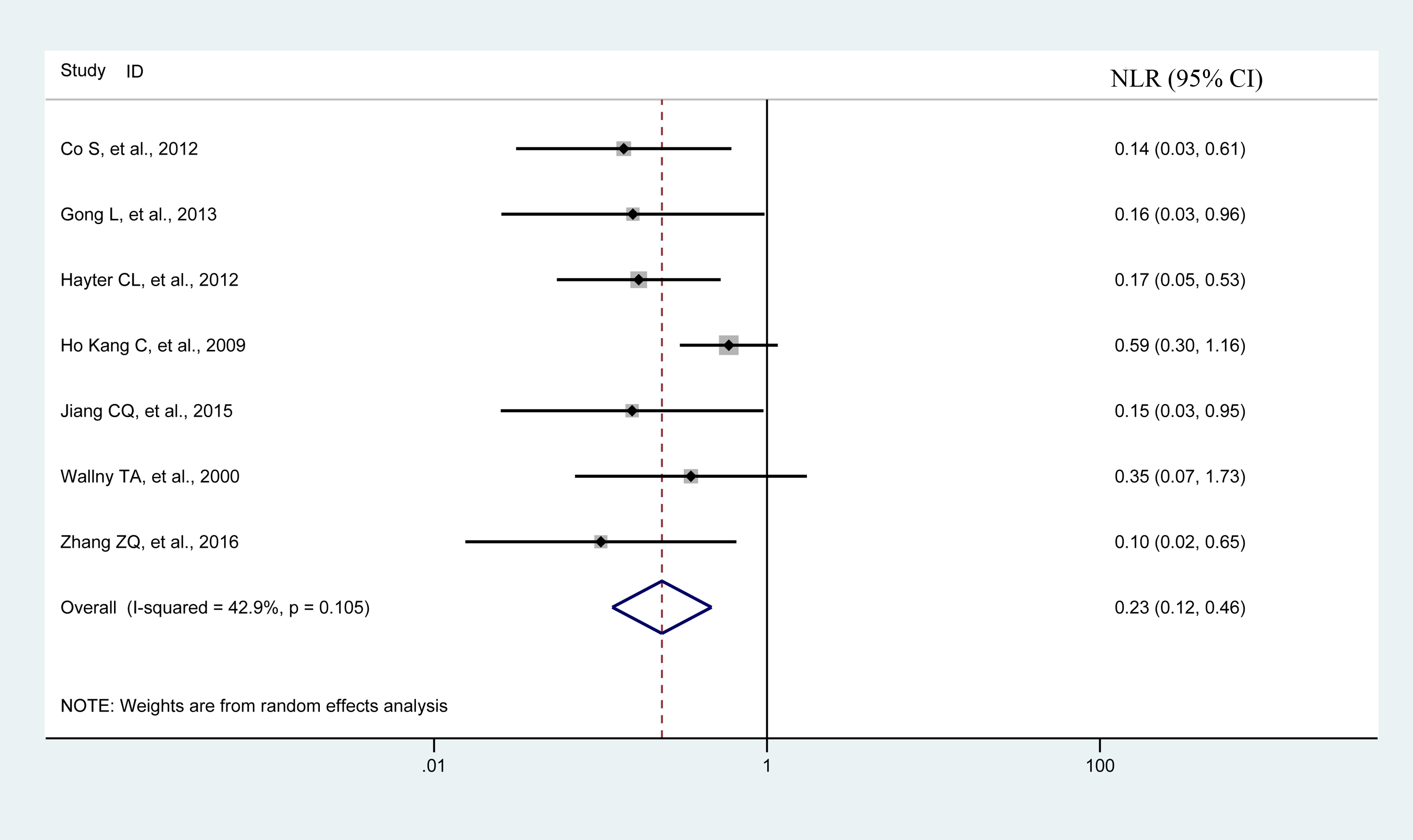

Supplement: Supplementary Figure S2 — Forest plots of the pooled diagnostic odds ratio (DOR) to diagnose partial-thickness rotator cuff tears with the corresponding 95% confidence region. The diamond in the right of the central vertical line represents a higher DOR to diagnose any rotator cuff tear. DOR, diagnostic odds ratio; CI, confidence interval. [file Image2.tiff]

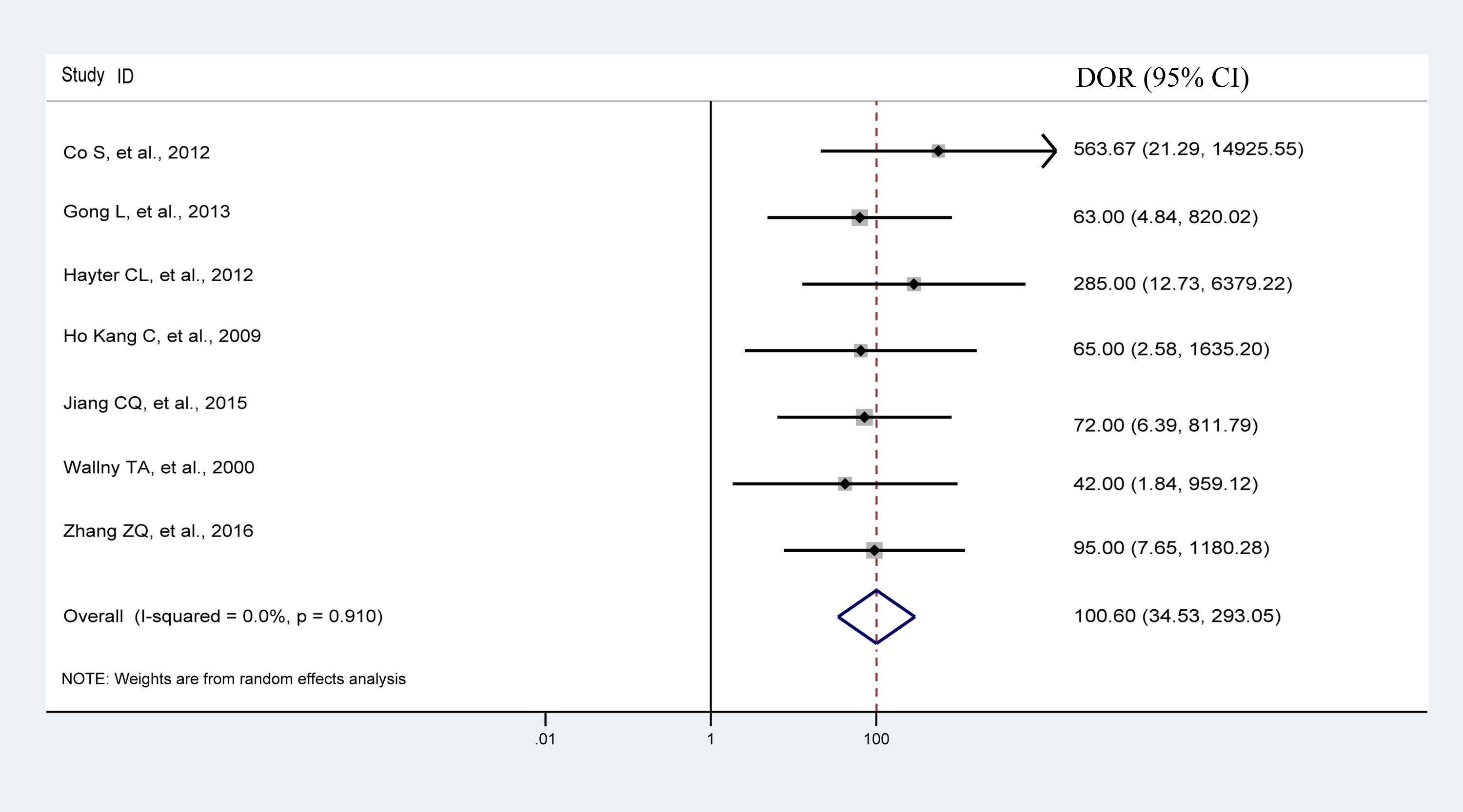

Supplement: Supplementary Figure S3 — Forest plots of the pooled PLR (A) and NLR (B) to diagnose full-thickness rotator cuff tear with the corresponding 95% confidence region. The diamond in the right of the central vertical line represents a higher PLR or NLR to diagnose any rotator cuff tear. NLR, negative likelihood ratio; PLR, positive likelihood ratio; CI, confidence interval. [file Image3.tiff]

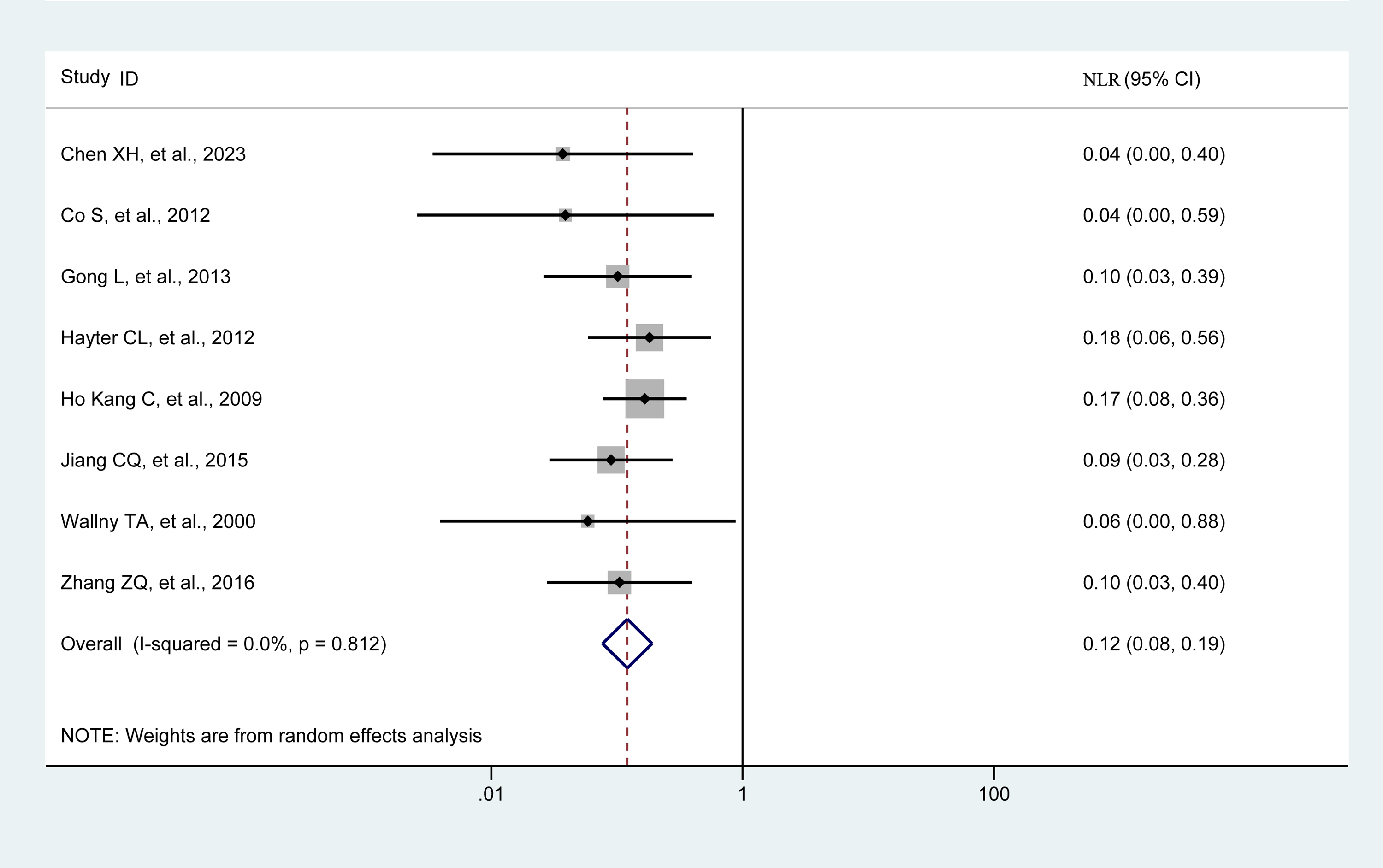

Supplement: Supplementary Figure S4 — Forest plots of the pooled diagnostic odds ratio (DOR) to diagnose full-thickness rotator cuff tears with the corresponding 95% confidence region. The diamond in the right of the central vertical line represents a higher DOR to diagnose any rotator cuff tear. DOR, diagnostic odds ratio; CI, confidence interval. [file Image4.tiff]

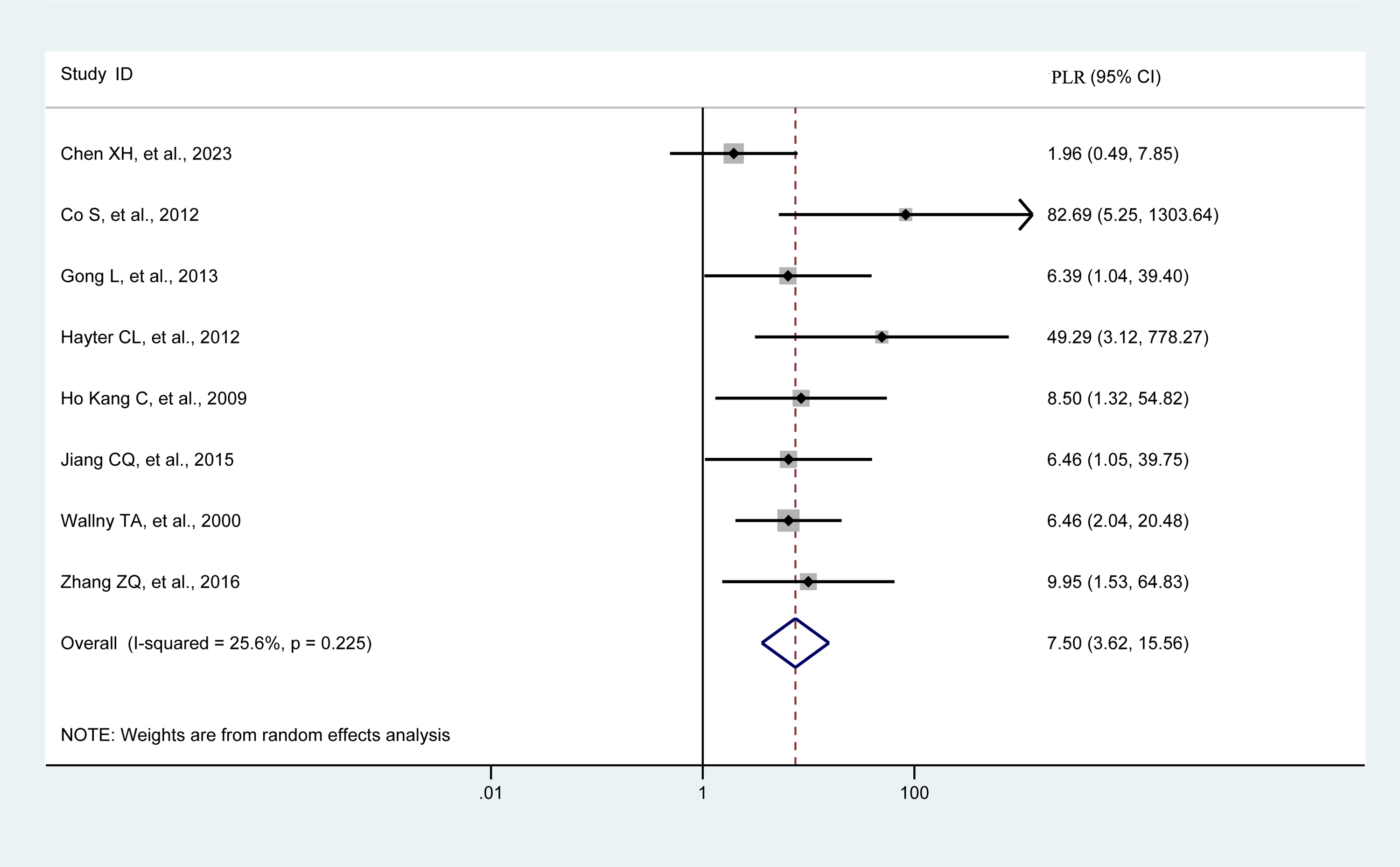

Supplement: Supplementary file 5 [file Image5.tiff]

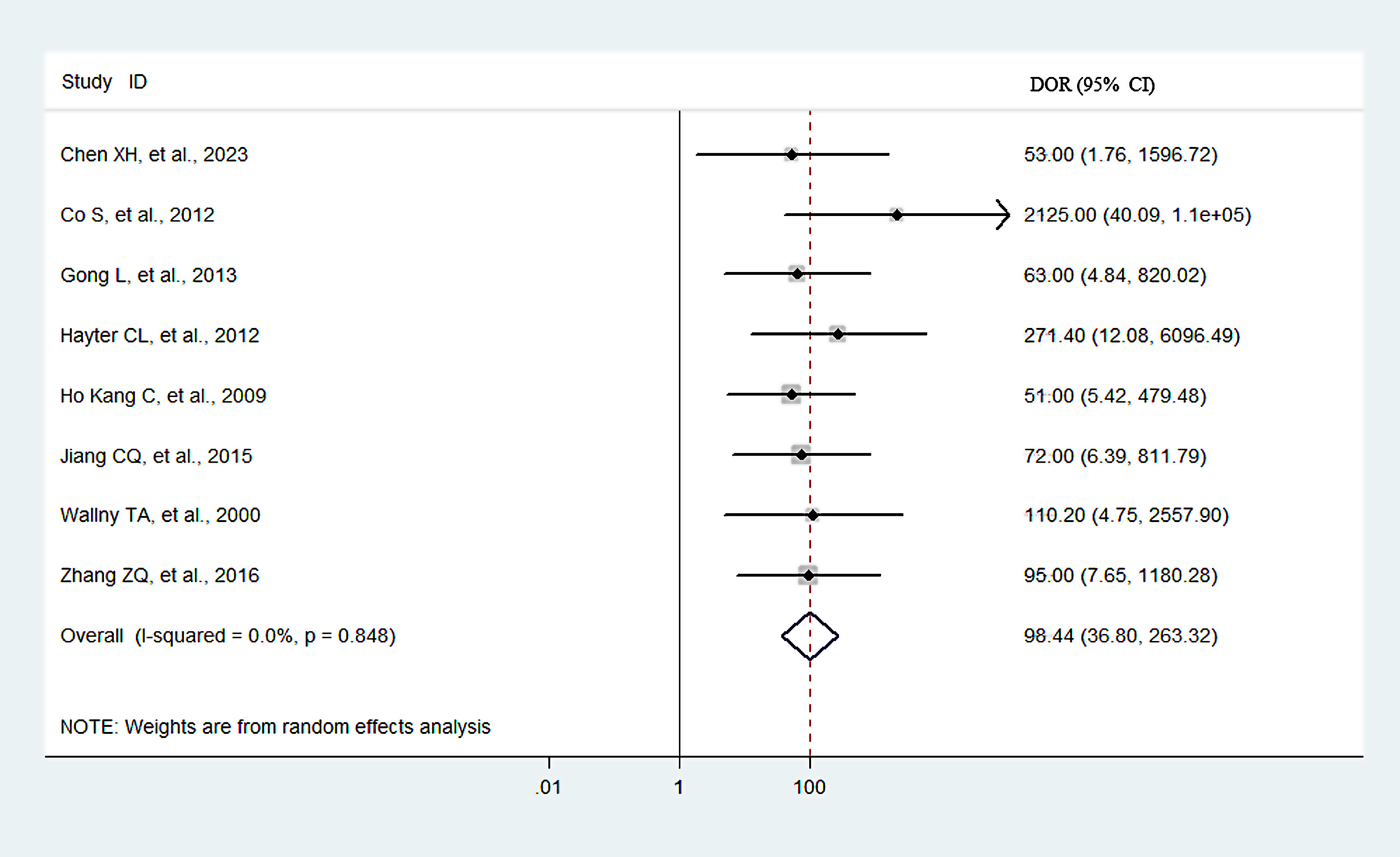

Supplement: Supplementary file 6 [file Image6.tiff]
